# CLOCK gene variation is associated with incidence of type-2 diabetes and cardiovascular diseases in type-2 diabetic subjects: dietary modulation in the PREDIMED randomized trial

**DOI:** 10.1186/s12933-015-0327-8

**Published:** 2016-01-07

**Authors:** Dolores Corella, Eva. M. Asensio, Oscar Coltell, José V. Sorlí, Ramón Estruch, Miguel Ángel Martínez-González, Jordi Salas-Salvadó, Olga Castañer, Fernando Arós, José Lapetra, Lluís Serra-Majem, Enrique Gómez-Gracia, Carolina Ortega-Azorín, Miquel Fiol, Javier Díez Espino, Andrés Díaz-López, Montserrat Fitó, Emilio Ros, José M. Ordovás

**Affiliations:** Department of Preventive Medicine and Public Health, Genetic and Molecular Epidemiology Unit, School of Medicine, University of Valencia, Blasco Ibañez, 15, 46010 Valencia, Spain; CIBER Fisiopatología de la Obesidad y Nutrición, Instituto de Salud Carlos III, Madrid, Spain; Department of Computer Languages and Systems, School of Technology and Experimental Sciences, Universitat Jaume I, Castellón, Spain; Department of Internal Medicine, Hospital Clinic, IDIBAPS, Barcelona, Spain; The PREDIMED (Prevención con Dieta Mediterránea) Research Network (RD 06/0045), Instituto de Salud Carlos III (ISCIII), Madrid, Spain; Department of Preventive Medicine and Public Health, University of Navarra, Pamplona, Navarra Spain; Human Nutrition Unit, Biochemistry and Biotechnology Department, Faculty of Medicine and Health Sciences, IISPV, University Rovira i Virgili, Reus, Spain; Cardiovascular Risk and Nutrition Unit, Hospital del Mar Institut for Medical Research (IMIM), Barcelona, Spain; Department of Cardiology, University Hospital of Araba, Vitoria, Spain; Department of Family Medicine, Research Unit, Distrito Sanitario Atención Primaria Sevilla, Seville, Spain; Research Institute of Biomedical and Health Sciences, University of Las Palmas de Gran Canaria, Las Palmas de Gran Canaria, Spain; Department of Epidemiology, School of Medicine, University of Malaga, Malaga, Spain; Palma Institute of Health Research (IdISPa), Hospital Son Espases, Palma de Mallorca, Spain; Atención Primaria, Servicio Navarro de Salud-Osasunbidea, Pamplona, Navarra Spain; Navarra Institute for Health Research (IdiSNA), Pamplona, Navarra Spain; Lipid Clinic, Endocrinology and Nutrition Service, Institut d’Investigacions Biomèdiques August Pi Sunyer (IDIBAPS), Hospital Clinic, Barcelona, Spain; Department of Cardiovascular Epidemiology and Population Genetics, Centro Nacional de Investigaciones Cardiovasculares (CNIC), Madrid, Spain; IMDEA Alimentación, Madrid, Spain; Nutrition and Genomics Laboratory, JM-USDA Human Nutrition Research Center on Aging at Tufts University, Boston, MA USA

**Keywords:** *CLOCK* gene, Diabetes, Cardiovascular diseases, Stroke, Mediterranean diet

## Abstract

**Background:**

Circadian rhythms regulate key biological processes influencing metabolic pathways. Disregulation is associated with type 2 diabetes (T2D) and cardiovascular diseases (CVD). Circadian rhythms are generated by a transcriptional autoregulatory feedback loop involving core clock genes. *CLOCK* (circadian locomotor output cycles protein kaput), one of those core genes, is known to regulate glucose metabolism in rodent models. Cross-sectional studies in humans have reported associations between this locus and obesity, plasma glucose, hypertension and T2D prevalence, supporting its role in cardiovascular risk. However, no longitudinal study has investigated the association between *CLOCK* gene variation and T2D or CVD incidence. Moreover, although in a previous work we detected a gene-diet interaction between the *CLOCK*-rs4580704 (C > G) single nucleotide polymorphism (SNP) and monounsaturated (MUFA) intake on insulin resistance, no interventional study has analyzed gene-diet interactions on T2D or CVD outcomes.

**Methods:**

We analyzed the association between the *CLOCK*-rs4580704 SNP and incidence of T2D and CVD longitudinally in 7098 PREDIMED trial (ISRCTN35739639) participants after a median 4.8-year follow-up. We also examined modulation by Mediterranean diet (MedDiet) intervention (high in MUFA) on these associations.

**Results:**

We observed a significant association between the *CLOCK*-rs4580704 SNP and T2D incidence in n = 3671 non-T2D PREDIMED participants, with variant allele (G) carriers showing decreased incidence (dominant model) compared with CC homozygotes (HR: 0.69; 95 % CI 0.54–0.87; P = 0.002). This protection was more significant in the MedDiet intervention group (HR: 0.58; 95 % CI 0.43–0.78; P < 0.001) than in the control group (HR: 0.95; 95 % CI 0.63–1.44; P = 0.818). Moreover, we detected a statistically significant interaction (P = 0.018) between *CLOCK*-rs4580704 SNP and T2D status on stroke. Thus, only in T2D subjects was *CLOCK*-rs4580704 SNP associated with stroke risk, G-carriers having decreased risk (HR: 0.61; 95 % CI 0.40–0.94; P = 0.024 versus CC) in the multivariable-adjusted model.

**Conclusions:**

In agreement with our previous results showing a protective effect of the G-allele against hyperglycemia, we extended our findings by reporting a novel association with lower T2D incidence and also suggesting a dietary modulation. Moreover, we report for the first time an association between a *CLOCK* polymorphism and stroke in T2D subjects, suggesting that core clock genes may significantly contribute to increased CVD risk in T2D.

**Electronic supplementary material:**

The online version of this article (doi:10.1186/s12933-015-0327-8) contains supplementary material, which is available to authorized users.

## Background

The continued increase in the incidence of type 2 diabetes (T2D) has elicited the need to investigate in more depth and breadth the risk factors that contribute to this disease in order to have a more comprehensive vision of the process to illuminate the path towards more precise and effective preventive interventions [1–3]. In addition, and given the impact of T2D on cardiovascular diseases (CVD) [2, 4–8] we need to gain further knowledge about the factors driving the connection between T2D and CVD. In this regard, there is increasing evidence linking chronodisruption with metabolic disorders [9–12].

All living things have acquired, in the course of evolution, an internal circadian timing system so as to adapt to the rhythmically occurring daily changes in their environment [13, 14]. In humans, this circadian timing coordinates virtually all physiological processes encompassing the states of sleep and wakefulness, endocrine functions and cardiovascular activity [15, 17]. It has been shown that several aspects of the cardiovascular physiopathology and incidence of CVD events, such as myocardial infarction, ischemic and hemorrhagic stroke, have diurnal variation, peaking in the early morning hours [18–23]. Moreover, a higher risk of obesity, metabolic alterations and T2D has been found in shift workers [24–27] and adverse cardiometabolic effects (including postprandial glucose in a range typical of a prediabetes state) have been detected in subjects who underwent forced circadian misalignment [28].

The endogenous circadian timing system includes the suprachiasmatic nucleus (SNC) in the hypothalamus, as well as peripheral oscillators in the different organs [29]. The core molecular clock is composed of a transcription-translation feedback loop that oscillates with 24-h rhythmicity [30]. Several core clock genes are involved in the regulation of this system [14, 30]. Among them, the *CLOCK* (Circadian Locomotor Output Cycles Kaput) gene, a transcription factors from the positive limb of the molecular clock that forms a complex with BMAL1 (Brain and Muscle ARNT-Like 1), is one of the most relevant [14, 30, 31]. In murine models, mutations in the clock gene were first associated with glucose homeostasis [32] and later with obesity, hyperglycemia and hyperinsulinemia [33]. In humans, various *CLOCK* single nucleotide polymorphisms (SNPs) (i.e. rs1464490, rs3749474, rs4864584, rs4580704 and rs18012602) have also been associated with obesity [34–36], hyperglycemia and greater prevalence of T2D in cross-sectional studies [36, 37]. However, there has been no longitudinal study to analyze the influence of *CLOCK* SNPs on T2D incidence. The *CLOCK*-rs4580704 C > G, a tag SNP in linkage disequilibrium with other *CLOCK* SNPs, is one of the most relevant SNPs for these associations [35, 36]. In our previous work in the Genetics of Lipid Lowering Drugs and Diet Network (GOLDN) population, we observed that carriers of the minor allele (G) for the *CLOCK*-rs4580704 SNP presented lower weight, fasting-insulin and hyperglycemia risk [36].

Moreover, we detected a gene-diet interaction between the *CLOCK*-rs4580704 SNP and monounsaturated fatty acids (MUFA) intake in determining fasting glucose and insulin resistance in GOLDN [36]. When the MUFA intake was low, no differences were found for these parameters. However, when MUFA intake was high, minor allele carriers had significantly lower levels. We also suggested that this SNP could be relevant in modulating CVD risk [36]. However, the latter connection has not been specifically investigated. Bearing in mind that there may be a greater alteration in circadian rhythms in T2D [9–11, 16], we hypothesized that the effect of variation in clock genes on CVD risk might be greater in T2D subjects. Our aims, therefore, were as follows: (1) to analyze the association between the tag SNP *CLOCK*-rs4580704 and the incidence of T2D; (2) to analyze the association between the *CLOCK*-rs4580704 SNP and incidence of clinical CVD events depending on T2D status and; (3) to study whether a dietary intervention with the Mediterranean diet (MedDiet), very high in MUFA, within the framework of the PREDIMED trial, modulates these associations.

## Methods

The present study was conducted within the framework of the PREvención con DIeta MEditerránea (PREDIMED) trial, whose design has been described in detail elsewhere [38]. Briefly, the PREDIMED study is a large, multicenter, randomized and controlled clinical trial aimed at assessing the effects of the Mediterranean diet (MedDiet) on primary cardiovascular prevention [38, 39]. This study was registered at controlled-trials.com (http://www.controlledtrials.com/ISRCTN35739639). Participants were randomized to one of three interventions: a MedDiet supplemented with extra virgin olive oil (EVOO), a MedDiet supplemented with mixed nuts, or advice on a low-fat diet (control diet). Here we included 7098 participants from whom DNA was isolated, the polymorphism determined, and who had valid data for the main clinical variables analyzed. The completion date of this study was December 2010 and the total number of randomized subjects was 7447. The 7098 participants included in the present study did not differ in the main characteristics from those of the total cohort. Additional file 1: Figure S1 shows the CONSORT flowchart of the trial for the primary outcomes. Details of the PREDIMED trial including sample size calculations and interim analysis have been fully described elsewhere [38, 39]. Briefly, From October 2003 physicians in Primary Care Centers selected high cardiovascular risk participants. Eligible were community-dwelling persons (55–80 years for men; 60–80 years for women) who met at least one of two criteria: T2D or 3 or more cardiovascular risk factors as previously detailed [38, 39]. Taking into account that no significant differences in CVD incidence were found between the MedDiet groups [39], data were analyzed pooling together the MedDiet intervention groups versus the control group.

A detailed description of the nutritional interventions has been provided elsewhere [39]. The Institutional Review Board/Ethics Committee of each participating center approved the study protocol. All participants provided written informed consent. Participants were followed up for a median of 4.8 years (interquartile range 2.8–5.8 years). Incidence of CVD as primary outcome and incidence of T2D in non-T2D participants (as secondary study outcome) were assessed.

### Demographic, clinical, anthropometric and dietary measurements

The baseline examination included assessment of standard cardiovascular risk factors, medication use, socio-demographic factors and lifestyle variables by validated questionnaires [38]. Food consumption was determined by a validated semi-quantitative food frequency questionnaire (FFQ) [40] and energy and nutrient intake was derived. For dietary intake obtained by FFQ, n = 7040 subjects were analyzed after exclusion of n = 58 subjects with invalid data. Adherence to MedDiet at baseline was assessed by a validated 14-item questionnaire [41]. Physical activity was estimated by the Minnesota Leisure-Time Physical Activity Questionnaire, as previously reported [38].

Weight and height were measured with calibrated scales and a wall-mounted stadiometer, respectively. BMI and the waist-to-height ratio were calculated. Blood pressure and heart rate were measured in triplicate using a validated semiautomatic oscillometer (Omron HEM-705CP, Hoofddorp, The Netherlands) with a 5-minute interval between each measurement with the patients seated and at rest in a peaceful setting. The means of these measurements were calculated. Hypertension was defined as having blood pressure ≥140/90 mm Hg or treatment with an antihypertensive medication) and dyslipidemia was defined as having a high plasma LDL-C concentration (≥160 mg/dL or lipid-lowering therapy), a low plasma HDL-cholesterol concentration (≤40 in men and ≤50 mg/dL in women).

### Biochemical determinations, DNA extraction and genotyping

At baseline, blood samples were obtained after an overnight fast. Fasting glucose and lipids were measured as previously described [42]. Biochemical data were available for the following number of participants with valid genotype data for the rs4580704 SNP: fasting glucose (n = 6716), total cholesterol (n = 6834), HDL cholesterol (n = 6753), LDL cholesterol (n = 6698), and triglycerides (n = 6795). Genomic DNA was extracted from buffy-coat and the *CLOCK*-rs4580704 (C > G) polymorphism, was genotyped on a 7900HT Sequence Detection System (Applied Biosystems, Foster City, CA, USA) using a fluorescent allelic discrimination TaqManTM assay (n = 7098 valid genotypes). Genotype frequencies did not deviate from Hardy–Weinberg equilibrium expectations (P = 0.145).

### Outcome ascertainment

#### CVD outcomes

The primary endpoint was the occurrence of the first major CVD events and comprised a composite endpoint including myocardial infarction, stroke or cardiovascular death [38, 39]. We used four sources of information to identify end-points: (1) repeated contacts with participants; (2) family physicians; (3) yearly review of medical records; and (4) consultation of the National Death Index. The end-point adjudication committee, whose members were blind to treatment allocation, examined all medical records related to end-points. Only end-points confirmed by the adjudication committee that occurred between October 1, 2003, and December 1, 2010 were included in the analyses (n = 265). The criteria for adjudicating primary outcomes are detailed elsewhere [39].

#### T2D outcomes

Clinical diagnosis of prevalent T2D was an inclusion criteria of the PREDIMED study as previously reported [38], and these subjects (n = 3427) were considered as prevalent cases of T2D. Incidence of T2D was a pre-specified secondary outcome of the PREDIMED trial [38]. New-onset diabetes during follow-up was diagnosed using the American Diabetes Association criteria, namely fasting plasma glucose levels ≥7.0 mmol/L (≥126.1 mg/dL) or 2-hour plasma glucose levels ≥11.1 mmol/L (≥200.0 mg/dL) after a 75-g oral glucose load, as previously reported [43]. A review of all medical records of participants was completed yearly in each center by physician-investigators who were blinded to the intervention. When new-onset T2D cases were identified on the basis of a medical diagnosis reported in the medical charts or on a glucose test during routine biochemical analyses (conducted at least once per year), these reports were sent to the PREDIMED Clinical Events Committee [38], whose members were also blinded to dietary group or the *CLOCK* genotype. All confirmed cases that occurred between October 1, 2003 and December 1, 2010 were included in the present analysis (n = 286).

### Statistical analyses

Data were analyzed at baseline and longitudinally after a median of 4.8 y of follow-up. We used ANOVA tests to compare the means of the continuous variables at baseline for the *CLOCK*-rs4580704 C > G genotypes. Association with fasting glucose at baseline was also estimated in a dominant model adjusted for covariates. Chi square tests were used to test differences in percentages. The *CLOCK*-rs4580704 SNP was first tested as following a general genetic model, including the three genotype categories (the CC genotype was considered the reference category) in order to check the model assumptions. After, if similar effects for CG and GG subjects were observed, dominant models grouping carriers of the variant G-allele were analyzed and presented as the selected genetic model. Incidence of T2D was estimated in non-T2D participants at baseline (n = 3671). To examine the association between the polymorphism and T2D incidence in non-T2D subjects at baseline, we used Cox regression models with the length of follow-up as the primary time variable. Follow-up time was calculated from the date of enrolment to the date of diagnosis of T2D for cases, and to the date of the last visit or the end of the follow-up period (December 1, 2010 for non-cases), or the date at death, depending on whichever came first. Hazard ratios (HR) with 95 % confidence intervals (CI) for the *CLOCK*-rs4580704 C > G SNP, were computed. Analyses were based on the intention-to-treat principle. In multivariable model 1 (basic model) we adjusted for sex, age, center and intervention group. Afterwards an additional control for more potential confounders such as BMI, smoking, drinking, physical activity, medications (for hypertension, dyslipidemia), adherence to the MedDiet and total energy intake at baseline (model 2) was carried out. Moreover, to analyze the heterogeneity of genetic effects by the dietary intervention, we tested the statistical significance of the interaction term between the *CLOCK*-rs4580704 SNP (dominant model) and the dietary intervention (subjects in the two MedDiet groups were merged in a single category (MedDiet group) versus the control group) in the multivariable Cox regression model (models 1 and 2). Stratified analyses by dietary intervention group (MedDiet and control group) were additionally carried out and HR and 95 % CI for T2D were estimated in each stratum as indicated above. Kaplan–Meier survival curves (cumulative T2D-free survival) were plotted to estimate the probability of remaining free of T2D during follow-up depending on the *CLOCK*-rs4580704 genotype.

To examine the association between the *CLOCK*- rs4580704 SNP and CVD in the whole population (non-T2D and T2D subjects) and by T2D status (at baseline), we used Cox regression models with the length of follow-up as the primary time variable. Follow-up time was calculated from the date of enrolment to the date of diagnosis of CVD for cases, and to the date of the last visit or the end of the follow-up period (December 1, 2010 for non-cases), or the date at death, depending on whichever came first. Hazard ratios (HR) with 95 % confidence intervals (CI) for the *CLOCK*-rs4580704 SNP, were computed. Analyses were based on the intention-to-treat principle. In multivariable model 1 (basic model) we adjusted for sex, age, center and intervention group. Afterwards an additional control for more potential confounders such as BMI, smoking, drinking, physical activity, medications (hypertension, dyslipidemia, hyperglycemia), adherence to the MedDiet and total energy intake at baseline (model 2) was carried out as indicated above for T2D. The interaction term between diabetes status and the *CLOCK*-rs4580704 C > G SNP in determining total CVD (general genetic model) and specific causes of CVD (dominant model) was tested in the multivariable Cox regression model (models 1 and 2). In T2D subjects, we also estimated the association between the *CLOCK*-rs4580704 C > G SNP and stroke and myocardial infarction using multivariable Cox regression models. Additionally, for the stroke outcome we tested the potential heterogeneity of genetic effect (dominant model) of dietary intervention (MedDiet versus control group). Kaplan–Meier survival curves for stroke were also plotted. Statistical analyses were performed with the IBM SPSS Statistics version 22, NY. All tests were two-tailed and P values <0.05 were considered statistically significant.

## Results

**Fig. 1:**
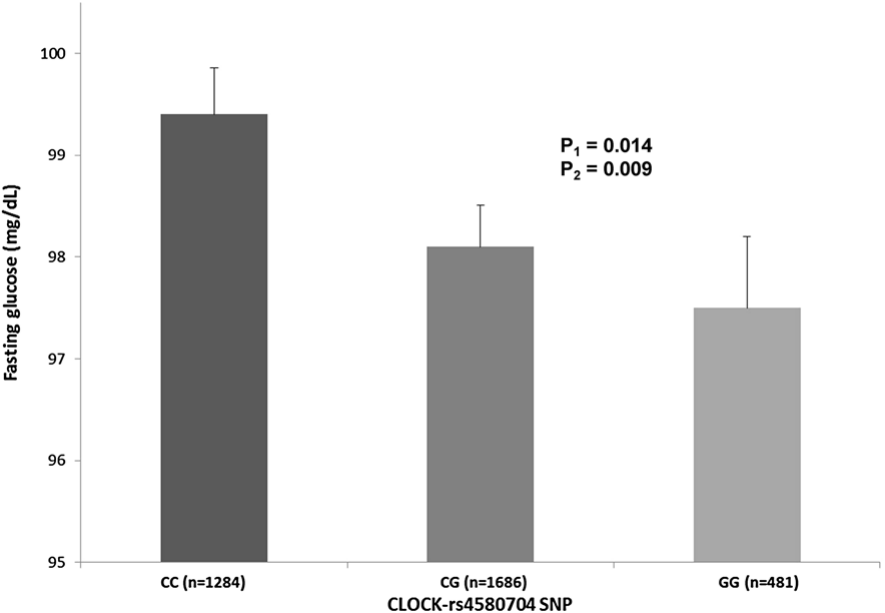
Association between the CLOCK-rs4580704 SNP and fasting glucose in non-T2D subjects at baseline. Means values and SE of means (*error bars*) are represented depending on the CLOCK genotypes. The P-values for the polymorphism were obtained in the dominant model for G-carriers vs CC subjects. *P*_*1*_ Unadjusted model. *P*_*2*_ Model adjusted for sex, age, field center and BMI

Additional file 1: Table S1 shows demographic, clinical, lifestyle and genetic characteristics of the studied population (n = 7098 subjects) at baseline depending on dietary intervention group. Prevalence of the *CLOCK*-rs4580704 C > G genotypes was as follows: CC (37.6 %), CG (48.1 %) and GG (14.3 %). No statistically significant differences of genotype prevalence among the dietary intervention groups was detected (P = 0.570). The minor allele frequency (MAF) for the G-variant allele was 0.38. General characteristics of participants by diabetes status are presented in Additional file 1: Table S2. Table 1 shows demographic, clinical and lifestyle characteristics of the study participants at baseline, depending on the *CLOCK*-rs4580704 C > G SNP. We observed statistically significant associations of the polymorphism with anthropometric variables (weight, BMI and waist circumference) at baseline. These parameters were lower in homozygous subjects for the variant G-allele. No statistically significant differences were detected for blood pressure, plasma lipid concentrations, fasting glucose, physical activity, total energy and macronutrient intake. An association was observed for adherence to the MedDiet, being slightly higher in carriers of the G-allele and this variable was further included in the multivariable adjusted model. By T2D status, the most relevant association was found with fasting glucose in non-T2D subjects at baseline (Fig. 1). Fasting plasma glucose concentrations were lower in carriers of the G-allele (P value in model 1 = 0.009). This association remained statistically significant even after additional adjustment for BMI (P value in model 2 = 0.014), suggesting independent effects of the associations.

**Table 1:** Demographic, clinical, lifestyle and genetic characteristics of the study participants at baseline according to the CLOCK-rs4580704 genotype

	CC (n = 2667)		CG (n = 3415)		GG (n = 1016)		P^1^
Age (years)	66.9	(6.2)	67.0	(6.2)	67.0	(6.3)	0.961
Weight (Kg)	76.9	(12.0)	77.1	(12.1)	75.4	(11.5)	<0.001
BMI (Kg/m^2^)	30.0	(3.8)	30.0	(3.9)	29.7	(3.8)	0.030
Waist circumference (cm)	100.5	(10.3)	100.6	(10.9)	99.3	(10.3)	0.002
Female sex n, %	1551	(58.2)	1932	(56.6)	587	(57.8)	0.444
Current smokers n, %	365	(13.7)	494	(14.5)	143	(14.1)	0.399
T2D prevalence n, %	1314	(49.3)	1616	(47.3)	497	(48.9)	0.291
*CLOCK*-*rs4580704 n,* *%*							0.570
MedDiet with EVOO	901	(33.8)	1208	(35.4)	339	(33.4)	
MedDiet with nuts	927	(34.8)	1078	(31.6)	328	(32.3)	
Control	839	(31.5)	1129	(33.1)	349	(34.4)	
SBP (mm Hg)	149.1	(20.8)	149.6	(20.7)	149.5	(21.0)	0.676
DBP (mm Hg)	83.5	(11.1)	83.5	(10.9)	83.0	(11.0)	0.389
Heart rate (bpm)	71.5	(11.1)	71.4	(11.3)	70.7	(10.8)	0.117
Total cholesterol (mg/dL)	209.9	(37.8)	211.2	(39.0)	210.0	(36.2)	0.417
LDL-C (mg/dL)	129.2	(33.7)	130.0	(34.0)	129.6	(32.7)	0.635
HDL-C (mg/dL)	53.8	(13.7)	53.6	(13.9)	54.5	(14.3)	0.224
Triglycerides (mg/dL)	137.5	(75.5)	137.6	(75.1)	132.6	(68.6)	0.109
Fasting glucose (mg/dL)	122.9	(41.8)	121.8	(41.8)	120.4	(39.1)	0.268
Energy intake (kcal/d)	2275	(603)	2281	(605)	2272	(605)	0.887
Total fat (g/d)	98.8	(30.3)	99.1	(30.6)	97.8	(29.9)	0.510
Saturated fat (g/d)	25.2	(9.2)	25.5	(9.3)	25.0	(8.9)	0.313
MUFA (g/d)	48.9	(16.0)	49.0	(16.0)	48.6	(16.0)	0.832
PUFA (g/d)	15.8	(6.9)	15.9	(7.2)	15.6	(6.6)	0.380
Proteins (g/d)	92.3	(22.8)	92.6	(23.2)	93.2	(23.5)	0.558
Carbohydrates (g/d)	239.6	(80.6)	239.5	(81.1)	239.9	(81.2)	0.987
Adherence to the MedDiet (points)^a^	8.6	(2.0)	8.7	(2.0)	8.7	(2.0)	0.037
Alcohol consumption (g/d)	8.3	(14.4)	8.6	(14.1)	8.4	(14.6)	0.718
Physical activity (METs-min/day)	232	(238)	227	(237)	243	(253)	0.218

Total indicates the maximum number of participants included with genotype data and demographic, anthropometric, adherence to MedDiet, physical activity and clinical variables. For dietary intake obtained by food-frequency questionnaires n = 7040 subjects were analyzed after exclusion of n = 58 subjects with invalid data. Biochemical data were available for fasting glucose (n = 6716 participants) total cholesterol (n = 6834 participants), HDL cholesterol (n = 6753 participants), LDL cholesterol (n = 6698 participants), and triglycerides (n = 6795 participants)

*MUFA* Monounsaturated fatty acids, *MedDiet* Mediterranean diet, *EVOO* extra virgin olive oil

^a^Based on a 14-point screener of adherence

^1^ *P* unadjusted P-value obtained in the ANOVA test

### Association between the CLOCK-rs4580704 polymorphism and incidence of T2D in non-T2D subjects: Interaction with dietary intervention

We analysed the association between the *CLOCK*-rs4580704 polymorphism and incidence of T2D in non-T2D subjects at baseline (n = 3671). Table 2 shows incidence rates and HR (95 % CI) for T2D varied by *CLOCK* genotype. As carriers of the G-allele presented similar incidence rates in the general genetic model, we pooled together these subjects (dominant model) and estimated T2D risk for G-carriers compared with CC homozygotes (reference category). G-allele carriers presented lower T2D risk than CC homozygous subjects (HR: 0.68; 95 % CI 0.54–0.86; P = 0.001). This protective association remained statistically significant (HR: 0.69; 95 % CI 0.54–0.87; P = 0.002) after additional adjustment for BMI and the other variables in model 2, suggesting an independent effect of *CLOCK*-rs4580704 polymorphism on incident T2D. Additional file 1: Figure S2 shows Kaplan–Meier curves for cumulative T2D-free survival by *CLOCK*-rs4580704 genotypes (dominant model) in non-T2D subjects over the 4.8 years median follow-up period.

**Table 2:** Incidence rate and hazard ratios (HR) for type-2 diabetes (T2D) depending on the CLOCK-rs4580704 in non-T2D subjects

CLOCK genotypes	Non-T2D subjects at baseline (n = 3671)								
	Cases	Person-y	Incidence rate^a^	Model 1			Model 2		
				HR	95 % CI	P value	HR	95 % CI	P value
Dominant model									
CC	130	6088.5	21.4	1.00	(Reference)		1.00	(Reference)	
CG + GG	156	10727.7	14.5	0.68	(0.54–0.86)	0.001	0.69	(0.54–0.87)	0.002

Model 1: adjusted for sex, age, center and dietary intervention group

Model 2: adjusted for variables in model 1 plus BMI, drinking, smoking, physical activity, medication (hypertension, dyslipemia and glucose), adherence to the Mediterranean Diet and total energy intake at baseline

^a^Crude incidence rates were expressed per 1000 person-years of follow-up

**Fig. 2:**
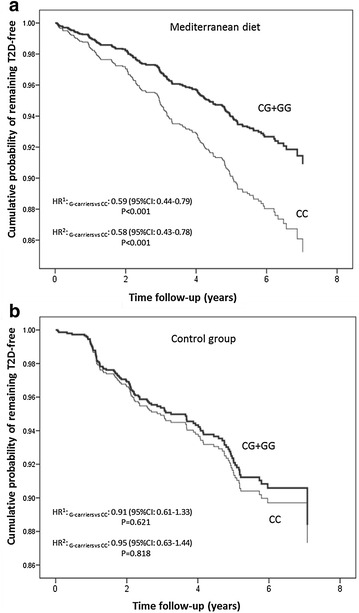
Cumulative T2D-free survival by *CLOCK*-rs4580704 genotypes in non-T2D subjects at baseline depending on the dietary intervention group.** a** Mediterranean diet groups (n = 2477); and **b** control group (n = 1194). Cox regression models with outcome of T2D incidence by the CLOCK-rs4580704 SNP (CC versus carriers of the G-allele) were multivariable adjusted for each stratum and the corresponding hazard ratios (HR) and 95 % CI were obtained in the multivariable adjusted models. CC subjects were considered the reference category and HR for G-carriers versus CC were estimated. *HR* ^*1*^ model adjusted for sex, age and field center. *HR* ^*2*^ Models additionally adjusted for BMI, drinking, smoking, physical activity, medications and total energy intake at baseline. P for interaction between the *CLOCK* SNP (as dominant) with dietary intervention (MedDiet vs control group) = 0.052 in model 2

When we analysed the modulation of the *CLOCK*-rs4580704 polymorphism and incidence of T2D depending on the dietary intervention, we obtained a *P* value for the interaction term (P = 0.052 in model 2) suggestive such heterogeneity (P < 0.1). Figure 2 shows cumulative T2D-free survival by *CLOCK*-rs4580704 genotypes (dominant model) in non-T2D subjects depending on the dietary intervention group [**A**: MedDiet groups (n = 2477); and **B**: control group (n = 1194)]. We observed that the protective association of the G-allele against T2D incidence was stronger and of higher statistical significance in the MedDiet intervention group (HR: 0.58; 95 % CI 0.43–0.78; P < 0.001) than in the control group (HR: 0.95; 95 % CI 0.63–1.44; P = 0.818) in model 2.

### Association between the CLOCK-rs4580704 polymorphism and incidence of CVD. Interaction with T2D status and modulation by dietary intervention

Additional file 1: Table S3 shows incidence rates of total CVD and HR and 95 % CI depending on the *CLOCK* polymorphism (general genetic model) in the whole population (T2D and non-TD2 subjects) and stratified by T2D. No similar effects were noted for CG and GG and the dominant model was not selected for total CVD. We detected a statistically significant interaction between T2D status and the *CLOCK*-rs4580704 SNP (P = 0.019 in the multivariable adjusted model 2) on total CVD incidence. The polymorphism was significantly associated with CVD incidence in T2D subjects (P = 0.039 in model 2) and no significant association was detected in non-T2D subjects (P = 0.649 in model 2). Thus, in the general genetic model (CC as reference category), we observed protective association between the GG genotype and CVD incidence (HR for GG versus CC = 0.48; 95 % CI 0.27–0.88; P = 0.016) in model 2 for T2D subjects. However, no significant association was observed for the GG genotype in non-T2D subjects (P = 0.370).

On analysing specific causes of CVD (Table 3), we obtained a significant interaction between the *CLOCK*-rs4580704 SNP and T2D status on stroke incidence (P for interaction: 0.018 in the dominant model 2), suggesting more specific effects for this outcome than for myocardial infarction. We observed a significant association between the *CLOCK* polymorphism and stroke in T2D, and no significant association was detected for myocardial infarction. For stroke, a protective association was observed in T2D G-allele carriers (HR: 0.61; 95 % CI 0.40–0.94; P = 0.024). Additionally, Additional file 1: Figure S3 shows cumulative stroke-free survival by *CLOCK*-rs4580704 genotypes (three categories) in T2D subjects. In non-T2D, the CLOCK SNP was no associated neither with stroke (HR: 1.59; 95 % CI 0.83–3–08; P = 0.165 nor with myocardial infarction (HR: 0.95; 95 % CI 0.48–1.89; P = 0.906).

**Table 3:** Incidence rate and hazard ratios (HR) for stroke and myocardial infarction depending on the CLOCK-rs4580704 in T2D subjects at baseline (n = 3427)

CLOCK genotypes	Cases	Person-y	Incidence rate^a^	Model 1			Model 2		
				HR	95 % CI	P value	HR	95 % CI	P value
Stroke									
Dominant model									
CC	43	5780.3	7.4	1.00	(Reference)		1.00	(Reference)	
CG + GG	43	9306.9	4.6	0.61	(0.40–0.93)	0.022	0.61	(0.40–0.94)	0.024
P (interaction CLOCK × T2D status)						0.016			0.018
Myocardial infraction									
Dominant model									
CC	24	5479.4	4.4	1.00	(Reference)		1.00	(Reference)	
CG + GG	40	9057.8	4.4	0.99	(0.60–1.65)	0.982	1.01	(0.61–1.70)	0.941
P (interaction CLOCK × T2D status)						0.906			0.922

Model 1: adjusted for sex, age, center and dietary intervention group

Model 2: adjusted for variables in model 1 plus BMI, drinking, smoking, physical activity, medication (hypertension, dyslipemia and glucose), adherence to the Mediterranean Diet and total energy intake at baseline

P for interaction terms between the CLOCK SNP (as dominant) with T2D in determining stroke or myocardial infarction were estimated in the corresponding multivariable models in the whole population

^a^Crude incidence rates were expressed per 1000 person-years of follow-up

Finally, we tested the modulation by the dietary intervention of the association between the *CLOCK*-rs4580704 SNP and stroke in T2D subjects. Additional file 1: Table S4 shows detailed information on stroke incidence rates, HR and 95 % CI by genotypes and intervention groups (MedDiet and control groups). Additional file 1: Table S3 shows additional information in the two MedDiet intervention groups. Although a higher protective association against stroke is apparent for G-carriers in the MedDiet group in comparison with the control group, the interaction term between the SNP and diet did not reach the statistical significance (P = 0.439 in model 2) and the heterogeneity by diet is not confirmed.

## Discussion

In this longitudinal study, undertaken on 7098 participants of the PREDIMED trial, we have been able to demonstrate the relevance of the *CLOCK*-rs4580704 SNP in the incidence of T2D and related processes, which finally lead to a higher incidence of CVD in T2D subjects, strengthening the connection between genetic variants in core clock genes, metabolic alterations and CVD risk. In agreement with our results, previous studies in humans analyzing this or other SNPs in linkage disequilibrium (rs1801260, rs3736544, rs4864548 and rs3749474) had already reported that the variant allele is associated with lower hyperglycemia and prevalence of T2D [36, 37]. However, given that cross-sectional studies may be confounded by other factors related with obesity and other phenotypes concurrently associated with T2D, our longitudinal study provides a higher evidence level for the contribution of the *CLOCK* gene to T2D incidence. Moreover, our results are supported by observations on murine models, given that mice with mutations resulting in under-expression of the clock gene presented obesity and hyperglycemia [32, 33]. This dual phenotype has been a limiting factor in establishing causality as it was not possible to tease apart whether the greater risk of diabetes in those mice [33] was directly related to the functionality of the clock gene or was mediated by obesity. Our study and previous epidemiological studies [34–36, 44] show that the *CLOCK*-rs4580704 SNP was also significantly associated with body-weight. However, through multivariable adjustment, we have been able to show that the associations of the *CLOCK*-rs4580704 SNP with fasting glucose and T2D incidence remain statistically significant after adjustment for BMI, supporting the notion that the observed effect may be additional to that of the association with obesity.

Furthermore, we have shown that the association between the *CLOCK*-rs4580704 SNP and T2D incidence is modulated by diet. Thus, in the stratified analysis by dietary intervention (MedDiet vs control diet), the protective association between the minor G-allele and T2D risk was enhanced in subjects allocated to the MedDiet intervention group being highly statistically significant. However, no significant protective effect in carriers of the G-allele was observed in subjects in the control (low-fat diet) group. Although, in our study, the gene-diet interaction term between the SNP and dietary intervention on T2D incidence was borderline significant (P = 0.052) at P < 0.05, taking into account that we can consider a P < 0.1 for the significance level of the interaction term as suggestive of interaction when some criteria of reliability are accomplished [45], our study fulfill these criteria to avoid false negative results and we considered this significance level (P < 0.1) to conduct the stratified analyses by MedDiet intervention groups. These criteria require that [45] the subgroup (dietary intervention) variable was measured at baseline and randomly distributed; we had an a priori hypothesis of the higher protective effect of the variant allele in the MedDiet intervention group (high MUFA); the direction of the subgroup effect was specified; a small number of hypotheses were tested and the size of the interaction effect was large. Our hypothesis of a higher protective effect against T2D of the variant allele (G) was based on our previous results obtained in the GOLDN study [36]. Therefore, our results support that the association between the *CLOCK*-rs4580704 SNP and T2D can be modulated by dietary intake, having the MedDiet pattern an increasing protective effect of the G-allele against the disease.

The dietary modulation of the CLOCK gene expression effects on metabolic phenotypes has been previously shown is animal models [11, 32, 33]. Moreover, in our previous study in a US white population we specifically reported a gene-diet interaction between the *CLOCK*-rs4580704 SNP and the contribution of MUFAs in the diet in determining fasting glucose concentrations and insulin resistance (HOMA-IR). Hence, when the MUFA intake (% of energy) was below the median (<13.2 %), no significant differences were found for plasma glucose concentrations and HOMA-IR between carriers and non-carriers of the G-allele. However, when MUFA intake was high (≥13.2 %), G-allele carriers had significantly lower plasma glucose concentrations and lower HOMA-IR values than did non-carriers. The MedDiet is precisely characterized by a high contribution of MUFAs (about 20 %), coming mainly from olive oil. In the intervention with MedDiet group, the MUFA content in the diet was significantly higher than in the low fat control group, as we described previously [39]. This high MUFA intake in the MedDiet intervention group could be one of the factors that may contribute to explain the higher protective effect against T2D incidence observed for G-carriers in the MedDiet intervention group but not in the control group in in agreement with our previous results obtained in the cross-sectional study carried out in the US population [36]. Furthermore, we can even hypothesize that the interaction effect between groups could be even higher and more significant if more differences in MUFA intake were reached (being a Mediterranean population, MUFA intake in the PREDIMED study is generally higher than in the US population). Although the mechanisms explaining the observed gene-diet modulation on T2D remain unknown, recent studies have suggested that DNA methylation may be an important mechanism to drive circadian clock plasticity [46, 47]. In support of this argument, we have shown, in Mediterranean subjects [48], that the percentage of methylation of certain CpG islands in the *CLOCK* gene were significantly associated with MUFA and PUFA intakes, adding preliminary evidence to the potential epigenetic dietary modulation, although this requires further additional work to be confirmed.

The polymorphism in the *CLOCK*-rs4580704 gene is located in an intron and its function is still unknown. However, it involves a tag SNP that is in linkage disequilibrium with other SNPs in the 3’UTR region that could be modulated by microRNAs. In our previous work focusing on this SNP [36], a specific bioinformatics analysis for *CLOCK*-rs4580704 SNP allowed us to affirm that SNP rs4580704 was predicted to produce an allele-specific CREM (cAMP responsive element modulator) binding site (C allele on forward strand binds CREM, G allele does not). In this bioinformatics analysis we used MAPPER [49] to identify potential allele-specific transcription factor binding sites and RNAfold [50] within the Vienna RNA package as previously detailed [36]. CREM has been shown to be responsible for circadian expression in the mouse of many genes that could also be implicated in T2D and CVD risk [51–53]. Either by itself, or as an indicator of another functional SNP, the variant G-allele of the *CLOCK*-rs4580704 SNP in our study is associated with lower fasting glucose concentrations in non-diabetics, lower T2D incidence and less CVD risk in T2D subjects. Bearing in mind that in murine clock mutant models [11, 32, 33], the reduction in clock gene expression is precisely associated with an opposite phenotype characterized by hyperglycemia, hyperinsulinemia and metabolic syndrome and considering that β cell clock gene ablation in mice caused severe glucose intolerance [10], the minor *CLOCK*-rs4580704 G-allele in humans would be associated with a higher conservation of gene functionality, i.e. in this case it would be the major allele which would provoke a situation of higher vulnerability to circadian adaptation and higher likelihood of chronodisruption. Carriers of the variant G-allele would be subjects that could better adapt to circadian disruption (i.e., deficient sleep, inadequate time of meals, shift work), or who maintain their rhythmicity of its processes with less alterations, presenting greater protection against obesity, T2D and future CVD.

Other SNPs in core circadian genes, such as BMAL1, that forms a complex with *CLOCK*, have been associated with T2D prevalence in some human studies and in murine models [11, 32, 54, 55] reinforcing the contribution of the circadian system to the etiology of T2D; however, no human study has reported an association between SNPs in core circadian genes and incidence of stroke as demonstrated in the current study. It is interesting to note that the association of the *CLOCK*-rs4580704 SNPs with stroke was only present in T2D subjects. This is compatible with a higher chronodisruption in these subjects, resulting in a higher CVD risk in a relatively short follow-up period (median follow-up approx. 5 years). Circadian variations in relevant risk factors for CVD such as insulin sensitivity, blood pressure, renal function, heart rate, platelet aggregability, fibrinolytic markers and levels, hormone concentrations etc. [9, 16, 17, 56–58] may explain the morning onset of myocardial infarction, stroke and other CVD clinical events [17, 18, 23]. These circadian variations may be more important in T2D patients and their dysregulation contributing to a higher CVD (mainly stroke) incidence in more susceptible individuals such as *CLOCK*-rs4580704 CC homozygous subjects (for whom our results suggest a lower flexibility). Although it is well known that T2D subjects have higher CVD risk than non-diabetic subjects [7, 8], a great heterogeneity has been described [59]. This heterogeneity in CVD risk is only partially understood but is a key consideration for our understanding of the nexus of T2D and CVD and for the development of individualized CVD risk reduction strategies. According to our results, we suggest circadian alterations due to functional genetic variants in core clock genes such as CLOCK as another factor that may contribute to this heterogeneity and higher risk of CVD (mainly, stroke) in those subjects. As measuring the processes of circadian dysregulation is complex, the *CLOCK*-rs4580704 SNP can be considered as a proxy for higher chronodisruption risk in CC subjects. Thus, this SNP will be of interest as an instrumental variant in future Mendelian Randomization in other cohorts analyzing CVD risk in T2D subjects.

## Conclusions

In conclusion, we have described for the first time an association between the *CLOCK*-rs4580704 SNP and incidence of T2D. These results are in agreement with previous knowledge obtained from animal and human cross-sectional studies. In addition we have found that this association with T2D can be modulated by dietary intake in the framework of a randomized controlled trial, with the MedDiet increasing the protective effects of the G-allele against T2D. Moreover, we have extended our finding also showing a novel association between the G-allele and protection against stroke in T2D subjects. Although our results highlight the contribution of the circadian system both on the incidence of T2D and CVD risk in T2D subjects it is presumable that the functional effects of CLOCK gene could be either circadian rhythm-dependent or independent. In the present study, as there is no assessments regarding the circadian rhythm of the study subjects, the suggested link between the CLOCK SNP and chronodisruption remains speculative, and more studies are needed to reveal the mechanisms behind these epidemiological associations.

## Additional file

10.1186/s12933-015-0327-8 Flow-chart. **Figure S2.** Cumulative T2D-free survival by *CLOCK*-rs4580704 genotypes depending on the dietary intervention group. **Figure S3.** Cumulative stroke-free survival by *CLOCK*-rs4580704 genotypes. **Table S1.** Characteristics of the PREDIMED study participants at baseline according to the dietary intervention groups. **Table S2.** Characteristics of the PREDIMED study participants at baseline according to the T2D status. **Table S3.** Incidence rate and hazard ratios (HR) for total cardiovascular diseases (CVD) depending on the *CLOCK*-rs4580704. **Table S4.** Incidence rate and hazard ratios (HR) for stroke depending on the *CLOCK*-rs4580704 in T2D subjects and stratified by dietary intervention.
