# Supplementary material for: CLOCK gene variation is associated with incidence of type-2 diabetes and cardiovascular diseases in type-2 diabetic subjects: dietary modulation in the PREDIMED randomized trial
Source: Cardiovasc Diabetol. 2016 Jan 7;15:4. doi: 10.1186/s12933-015-0327-8 (PMC4704407; doi:10.1186/s12933-015-0327-8)
Supplement: Supplementary file 1 — 10.1186/s12933-015-0327-8 Flow-chart. Figure S2. Cumulative T2D-free survival by CLOCK-rs4580704 genotypes depending on the dietary intervention group. Figure S3. Cumulative stroke-free survival by CLOCK-rs4580704 genotypes. Table S1. Characteristics of the PREDIMED study participants at baseline according to the dietary intervention groups. Table S2. Characteristics of the PREDIMED study participants at baseline according to the T2D status. Table S3. Incidence rate and hazard ratios (HR) for total cardiovascular diseases (CVD) depending on the CLOCK-rs4580704. Table S4. Incidence rate and hazard ratios (HR) for stroke depending on the CLOCK-rs4580704 in T2D subjects and stratified by dietary intervention. [file 12933_2015_327_MOESM1_ESM.pdf]

CLOCK gene variation is associated with incidence of type-2 diabetes and cardiovascular diseases in type-2 diabetic subjects: Dietary modulation in the PREDIMED randomized trial

Corella D et al.

SUPPORTING ONLINE MATERIAL

- **Figure S1**: ***Flow-chart for the primary outcome of the PREDIMED trial.***
- **Figure S2**: ***Cumulative T2D-free survival by CLOCK-rs4580704 genotypes in non-T2D subjects at baseline depending on the dietary intervention group***. A: Mediterranean diet groups (n=2,477); and B: control group (n=1,194). Cox regression models with outcome of T2D incidence by the CLOCK-rs4580704 SNP (CC versus carriers of the G-allele) were multivariable adjusted for each stratum and the corresponding hazard ratios (HR) and 95%CI were obtained in the multivariable adjusted models. CC subjects were considered the reference category and HR for G-carriers versus CC were estimated. HR1: model adjusted for sex, age and field center. HR2: Models additionally adjusted for BMI, drinking, smoking, physical activity, medications and total energy intake at baseline.
- **Figure S3**: ***Cumulative stroke-free survival by CLOCK-rs4580704 genotypes in T2D subjects at baseline (n=3,427)***. Cox regression models with outcome of stroke incidence by the CLOCK-rs4580704 SNP were multivariable adjusted. Hazard ratios (HR) and 95%CI were obtained in the multivariable adjusted models. CC subjects were considered the reference category and HR for G-carriers versus CC were estimated in the model adjusted for sex, age, field center, dietary intervention group, BMI, drinking, smoking, physical activity, medications and total energy intake at baseline.
- **Table S1**: ***Demographic, clinical, lifestyle and genetic characteristics of the PREDIMED study participants at baseline according to the dietary intervention groups***.
- **Table S2**: ***Demographic, clinical and lifestyle characteristics of the PREDIMED study participants at baseline according to the T2D status***.
- **Table S3**. ***Incidence rate and hazard ratios (HR) for total cardiovascular diseases (CVD) depending on the CLOCK-rs4580704. Total and stratified by T2D status at baseline***.
- **Table S4**. ***Incidence rate and hazard ratios (HR) for stroke depending on the CLOCK-rs4580704 in T2D subjects and stratified by dietary intervention***.

Figure S1: ***Flow-chart for the primary outcome of the PREDIMED trial.***

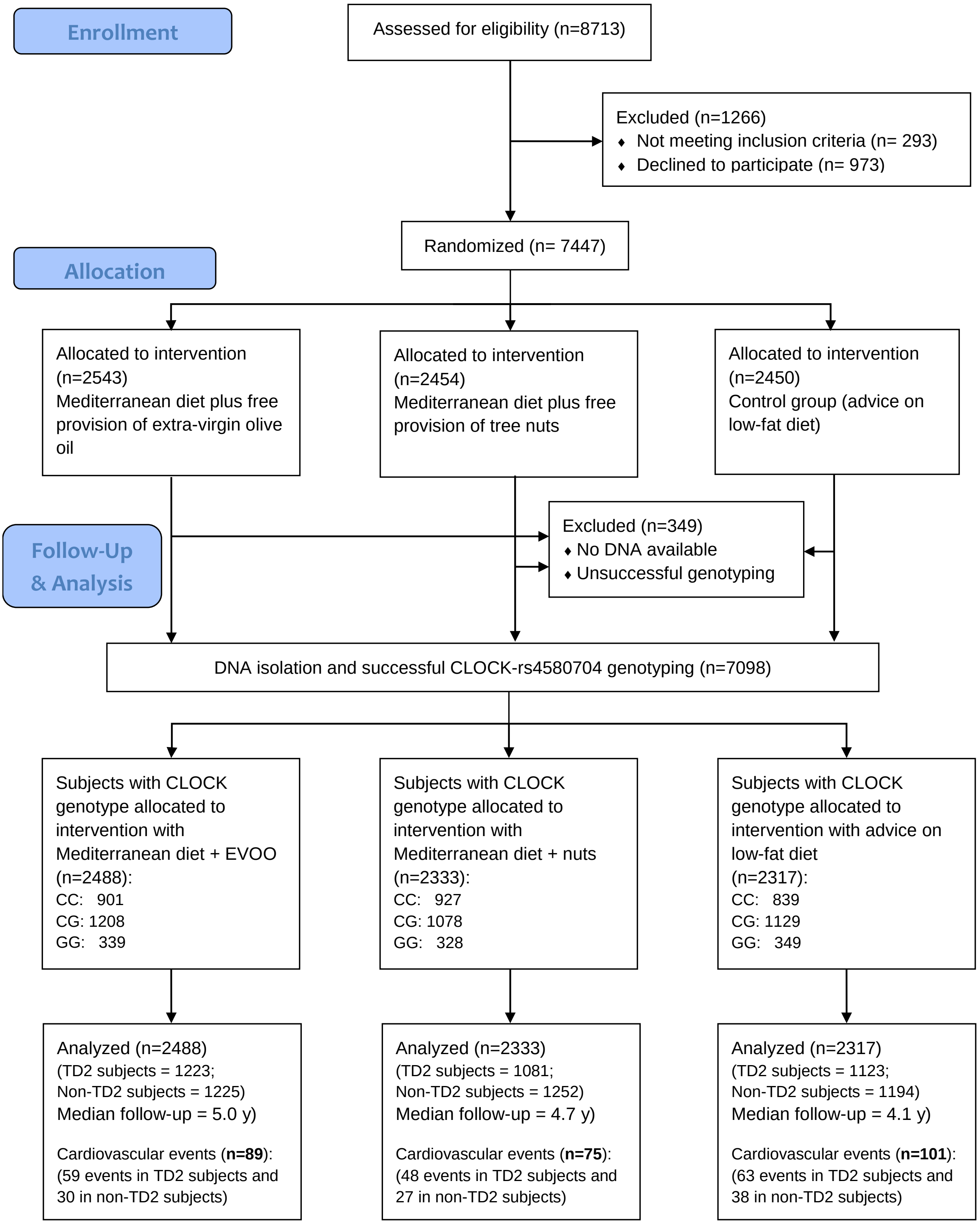

Table S1: Demographic, clinical, lifestyle and genetic characteristics of the PREDIMED study participants at baseline according to the dietary intervention groups[a]

	Total[b] (n=7,098)	MedDiet with EVOO (n=2,448)	MedDiet with Nuts (n=2,333)	Control group (n=2,317)
Age (years)	67.0 (6.2)	67.0 (6.1)	67.3 (6.1)	67.3 (6.3)
Weight (Kg)	76.8 (12.0)	76.6 (11.9)	77.0 (11.9)	77.0 (12.2)
BMI (Kg/m^2)	30.0 (3.8)	29.9 (0.1)	30.2 (0.1)	30.2 (4.0)
Waist circumference (cm)	100.4 (10.6)	100.1 (10.5)	100.8 (10.5)	100.8 (10.9)
Female sex : n, %	4070 (57.3)	1438 (53.6)	1381 (53.6)	1381 (59.6)
Current smokers: n, %	1002 (14.1)	343 (14.5)	320 (14.5)	320 (13.8)
Type 2 diabetes: n, %	3427 (48.3)	1223 (46.3)	1123 (46.3)	1123 (48.5)
Hypertension: n, %	5876 (82.8)	2007 (82.6)	1943 (82.6)	1943 (83.9)
Dyslipidemia: n, %	5131 (72.3)	1755 (73.2)	1669 (73.2)	1669 (72.0)
CLOCK- rs4580704: n, %				
CC	2667 (37.6)	901 (39.7)	839 (39.7)	839 (36.2)
CG	3415 (48.1)	1208 (46.2)	1129 (46.2)	1129 (48.7)
GG	1016 (14.3)	339 (14.1)	349 (14.1)	349 (15.1)
SBP (mm Hg)	149.4 (20.8)	148.5 (20.4)	150.1 (20.4)	150.1 (21.0)
DBP (mm Hg)	83 (11)	83 (11)	84 (11)	84 (11)
Heart rate (bpm)	71.4 (11.1)	71.3 (11.0)	71.6 (11.0)	71.6 (11.3)
Total cholesterol (mg/dL)	210.5 (38.2)	213.0 (37.5)	209.1 (37.5)	209.1 (38.9)
LDL-C (mg/dL)	129.6 (33.7)	131.7 (33.8)	128.5 (33.8)	128.5 (34.4)
HDL-C (mg/dL)	53.8 (13.9)	54.1 (13.9)	53.6 (13.9)	53.6 (13.8)
Triglycerides (mg/dL)	136.8 (74.4)	136.6 (76.4)	138.3 (76.4)	138.3 (73.4)
Fasting glucose (mg/dL)	122.1 (41.1)	122.6 (41.0)	122.9 (41.0)	122.9 (41.9)
Energy intake (kcal/d)	2277 (604)	2288 (607)	2224 (607)	2224 (599)
Total fat (g/d)	98.8 (30.4)	99.2 (30.0)	96.1 (30.0)	96.1 (30.7)
Saturated fat (g/d)	25.3 (9.2)	25.3 (9.1)	24.8 (9.1)	24.8 (9.3)
MUFA (g/d)	48.9 (16.0)	49.4 (15.6)	47.5 (15.6)	47.5 (16.4)
PUFA (g/d)	15.8 (7.0)	15.7 (7.1)	15.3 (7.1)	15.3 (6.8)
Proteins (g/d)	92.6 (23.1)	93.4 (22.9)	90.3 (22.9)	90.3 (22.3)
Carbohydrates (g/d)	239.6 (80.9)	240.4 (81.9)	236.3 (81.9)	236.3 (79.8)
Adherence to the MedDiet (points)[c]	8.7 (2.0)	8.8 (1.9)	8.4 (1.9)	8.4 (2.0)
Alcohol consumption (g/d)	8.5 (14.3)	8.6 (15.1)	7.5 (15.1)	7.5 (13.1)
Physical activity (METs-min/day)	231 (240)	232 (247)	214 (247)	214 (240)

[a]: Values are mean(SD) for continuous variables and number (%) for categorical variables. BMI indicates body mass index, MUFA, Monounsaturated fatty acids; MedDiet, Mediterranean diet; EVOO, extra virgin olive oil, SPB: Systolic blood pressure, DBP: Diastolic blood pressure.

MUFA, Monounsaturated fatty acids; MedDiet, Mediterranean diet; EVOO, extra virgin olive oil.

[b]: Total indicates the maximum number of participants included with genotype data and demographic, anthropometric, adherence to MedDiet, physical activity and clinical variables. For dietary intake obtained by food-frequency questionnaires n = 7040 subjects were analyzed after exclusion of n = 58 subjects with invalid data. Biochemical data were available for fasting glucose (n = 6716 participants) total cholesterol (n = 6834 participants), HDL cholesterol (n = 6753 participants), LDL cholesterol (n = 6698 participants), and triglycerides (n = 6795 participants).

[c]: Based on a 14-point screener of adherence.

Table S2: Demographic, clinical and lifestyle characteristics of the PREDIMED study participants at baseline according to the T2D status[a]

	Non-T2D subjects (n=3,671)	T2D subjects (n=3,427)	P[1]
Age (years)	66.6 (6.1)	67.4 (6.3)	<0.001
Weight (Kg)	76.7 (11.7)	76.9 (12.2)	0.419
BMI (Kg/m^2)	30.0 (3.6)	29.9 (4.1)	0.080
Waist circumference (cm)	99.6 (10.6)	101.2 (10.5)	<0.001
Female sex : n, %	2262 (61.6)	1808 (52.8)	<0.001
Current smokers: n, %	592 (16.1)	410 (12.0)	<0.001
CLOCK- rs4580704: n, %			0.291
CC	1353 (36.9)	1314 (38.3)	
CG	1799 (49.0)	1616 (47.2)	
GG	519 (14.1)	497 (14.5)	
SBP (mm Hg)	149.1 (20.7)	149.7 (20.9)	0.191
DBP (mm Hg)	84.5 (11.1)	82.2 (10.9)	<0.001
Heart rate (bpm)	70.0 (10.9)	72.8 (11.3)	<0.001
Total cholesterol (mg/dL)	219.6 (37.9)	200.8 (36.1)	<0.001
LDL-C (mg/dL)	137.1 (34.3)	121.6 (31.2)	<0.001
HDL-C (mg/dL)	55.8 (14.2)	51.7 (13.2)	<0.001
Triglycerides (mg/dL)	132.5 (69.6)	141.5 (79.0)	<0.001
Fasting glucose (mg/dL)	98.6 (16.7)	147.1 (44.5)	<0.001
Energy intake (kcal/d)	2322 (600)	2229 (605)	<0.001
Total fat (g/d)	99.0 (29.5)	98.6 (31.2)	0.552
Saturated fat (g/d)	25.2 (9.0)	25.5 (9.4)	0.154
MUFA (g/d)	49.3 (15.5)	48.6 (16.5)	0.076
PUFA (g/d)	15.8 (6.7)	15.9 (7.2)	0.824
Proteins (g/d)	92.0 (22.1)	93.2 (24.1)	0.030
Carbohydrates (g/d)	249.7 (82.0)	228.7 (78.3)	<0.001
Adherence to the MedDiet (points)[c]	8.7 (2.0)	8.6 (2.0)	<0.001
Alcohol consumption (g/d)	9.2 (14.9)	7.7 (13.6)	0.006
Physical activity (METs-min/day)	224 (225)	239 (255)	0.008

[a]: Values are mean(SD) for continuous variables and number (%) for categorical variables. BMI indicates body mass index, MUFA, Monounsaturated fatty acids; MedDiet, Mediterranean diet; EVOO, extra virgin olive oil, SPB: Systolic blood pressure, DBP: Diastolic blood pressure.

[1]: *P* unadjusted P-value for means comparison.

MUFA, Monounsaturated fatty acids; MedDiet, Mediterranean diet; EVOO, extra virgin olive oil.

[c]: Based on a 14-point screener of adherence.

Figure S2: ***Cumulative T2D-free survival by CLOCK-rs4580704 genotypes in non-T2D subjects at baseline depending on the dietary intervention group***. A: Mediterranean diet groups (n=2,477); and B: control group (n=1,194). Cox regression models with outcome of T2D incidence by the CLOCK-rs4580704 SNP (CC versus carriers of the G-allele) were multivariable adjusted for each stratum and the corresponding hazard ratios (HR) and 95%CI were obtained in the multivariable adjusted models. CC subjects were considered the reference category and HR for G-carriers versus CC were estimated. HR1: model adjusted for sex, age and field center. HR2: Models additionally adjusted for BMI, drinking, smoking, physical activity, medications and total energy intake at baseline.

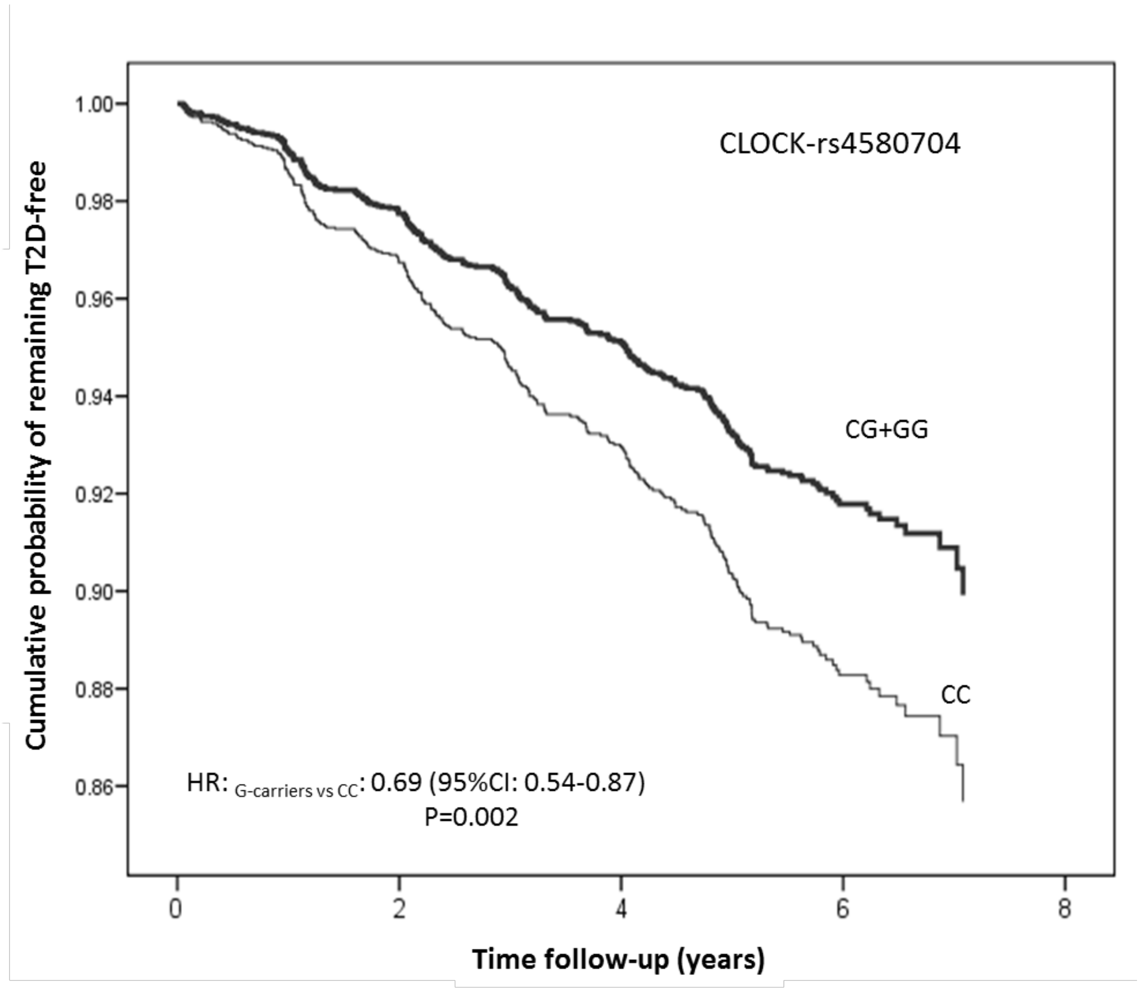

Table S3. Incidence rate and hazard ratios (HR) for total cardiovascular diseases (CVD) depending on the *CLOCK* -rs4580704. Total and stratified by T2D status at baseline

	Whole population (n = 7,098)								
				Model 1			Model 2		
CLOCK genotypes	CVD cases	person-y	Incidence rate*	HR	95% CI	P value	HR	95% CI	P value
General genetic model						0.235			0.366
CC	105	11414.8	9.2	1.00	(reference)		1.00	(reference)	
CG	131	14845.0	8.8	0.95	(0.74-1.24)	0.726	0.96	(0.75-1.25)	0.780
GG	29	4399.3	6.6	0.70	(0.47-1.06)	0.093	0.75	(0.49-1.13)	0.161
P (interaction CLOCK x T2D status)						0.017			0.019

	T2D subjects (n = 3,427)								
				Model 1			Model 2		
CLOCK genotypes	CVD cases	person-y	Incidence rate*	HR	95% CI	P value	HR	95% CI	P value
General genetic model						0.030			0.049
CC	73	5780.3	12.6	1.00	(reference)		1.00	(reference)	
CG	84	7126.6	11.8	0.92	(0.67-1.26)	0.590	0.92	(0.67-1.13)	0.587
GG	13	2180.3	6.0	0.45	(0.25-0.82)	0.008	0.48	(0.27-0.88)	0.016

	Non-T2D subjects (n = 3,671)								
				Model 1			Model 2		
General genetic model						0.738			0.649
CC	32	5642.0	5.7	1.00	(reference)		1.00	(reference)	
CG	47	7717.7	6.1	1.03	(0.65-1.61)	0.911	1.04	(0.66-1.64)	0.858
GG	16	2219.2	7.2	1.26	(0.69-2.29)	0.294	1.31	(0.72-2.42)	0.370

*: Crude incidence rates were expressed per 1000 person-years of follow-up

Model 1: Adjusted for sex, age, center and dietary intervention group.

Model 2: Adjusted for variables in model 1 plus BMI, diabetes, drinking, smoking, physical activity, medication (hypertension, dyslipemia and glucose), adherence to the Mediterranean Diet and total energy intake at baseline.

P for interaction terms between the *CLOCK* SNP (as general genetic model) with T2D were estimated in the corresponding multivariable model.

Figure S3: ***Cumulative stroke-free survival by CLOCK-rs4580704 genotypes in T2D subjects at baseline (n=3,427)***. Cox regression models with outcome of stroke incidence by the CLOCK-rs4580704 SNP were multivariable adjusted. Hazard ratios (HR) and 95%CI were obtained in the multivariable adjusted models. CC subjects were considered the reference category and HR for G-carriers versus CC were estimated in the model adjusted for sex, age, field center, dietary intervention group, BMI, drinking, smoking, physical activity, medications and total energy intake at baseline.

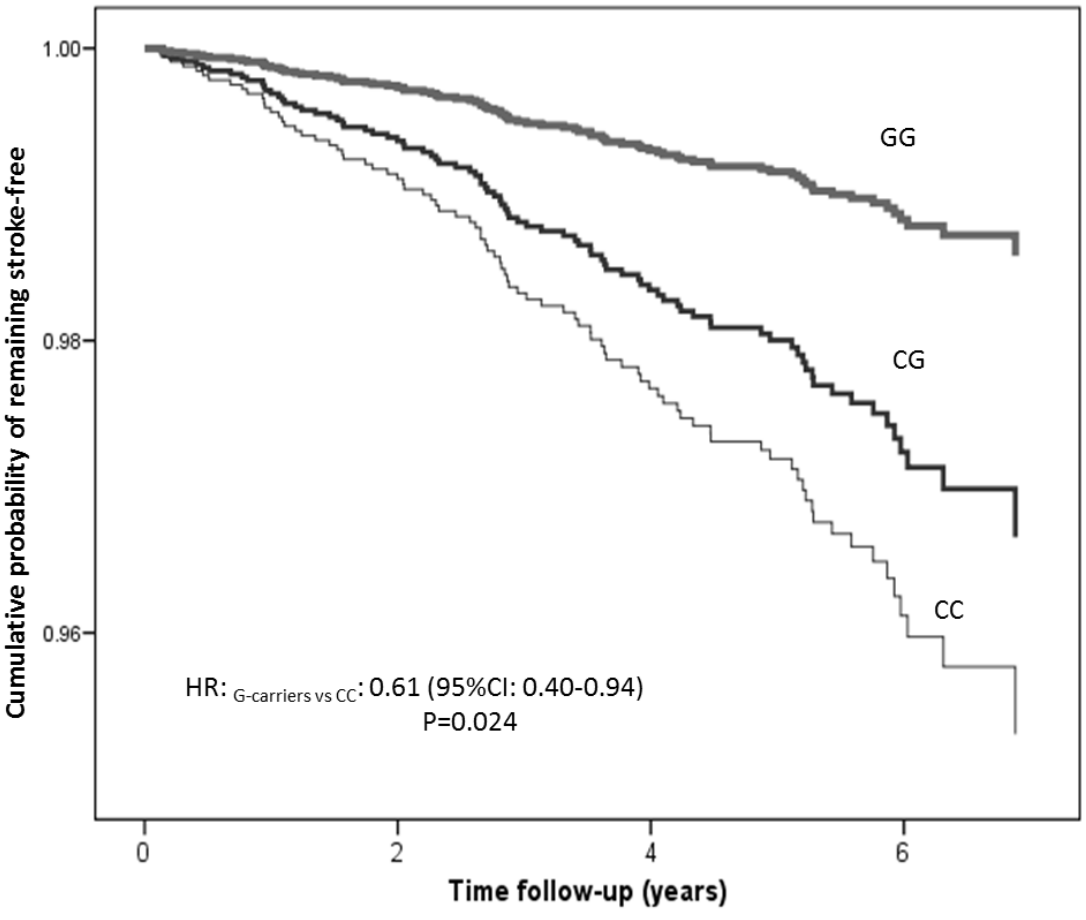

Table S4. Incidence rate and hazard ratios (HR) for stroke depending on the *CLOCK* -rs4580704 in T2D subjects and stratified by dietary intervention

	MedDiet (n = 2,304)								
				Model 1			Model 2		
CLOCK genotypes	cases	person-y	Incidence rate*	HR	95% CI	P value	HR	95% CI	P value
Dominant model						0.030			0.032
CC	27	4140.4	6.5	1.00	(reference)		1.00	(reference)	
CG + GG	22	6453.0	3.4	0.54	(0.31-0.94)	0.030	0.54	(0.31-0.95)	0.032
P (interaction CLOCK x MedDiet intervention)						0.470			0.439
	**Control group (n = 1,123)**								
				Model 1			Model 2		
Dominant model						0.306			0.335
CC	16	1639.8	9.8	1.00	(reference)		1.00	(reference)	
CG + GG	21	2856.1	7.4	0.71	(0.37-1.37)	0.306	0.72	(0.37-1.40)	0.335

*: Crude incidence rates were expressed per 1000 person-years of follow-up

MedDiet, Mediterranean diet; EVOO, extra virgin olive oil.

Model 1: Adjusted for sex, age, center and dietary intervention group.

Model 2: Adjusted for variables in model 1 plus BMI, drinking, smoking, physical activity, medication (hypertension, dyslipemia and glucose), adherence to the Mediterranean Diet and total energy intake at baseline.

P for interaction terms between the *CLOCK* SNP (as dominant) with dietary intervention (MedDiet vs Control group) were determined in the corresponding multivariable models.
